# Developing and Validating a Coding Scheme for Clinical Reasoning in History Taking Using Generative AI–Based Virtual Patients: Systematic Text Condensation Approach

**DOI:** 10.2196/84347

**Published:** 2026-04-13

**Authors:** Naping Chen, Luzhen Tang, Yang Liu, Changmin Lin, Zijian Li, Chujun Shi, Mengyu Xia, Dragan Gasevic, Danijela Gasevic, Jinbin Zheng, Yizhou Fan, Xinyu Li

**Affiliations:** 1 Department of Clinical Skills Training Center, Shantou University Medical College, Shantou, Guangdong, China; 2 Graduate School of Education, Peking University, 5th Yiheyuan Road, Beijing, 100871, China, 86 15210167528; 3 Office of Teaching Affairs, Shantou University Medical College, Shantou, Guangdong, China; 4 Faculty of Information Technology, Monash University, 25 Exhibition Walk, Clayton, Melbourne, VIC 3168, Australia; 5 School of Public Health and Preventive Medicine, Monash University, Melbourne, Australia; 6 Department of Cardiology, The First Affiliated Hospital of Shantou University Medical College, Shantou, Guangdong, China

**Keywords:** clinical reasoning, coding scheme, history taking, virtual patients, medical education, generative artificial intelligence

## Abstract

**Background:**

Effective history taking helps clinicians identify key symptoms and form accurate hypotheses. Generative artificial intelligence (GenAI)–based virtual patients (VPs) are increasingly used to simulate and practice history taking. However, there is currently no straightforward approach to effectively identify students’ clinical reasoning activities during these interactions, which limits the ability to provide instructional feedback.

**Objective:**

This study aims to develop and validate a coding scheme to identify medical students’ history-taking behaviors during interactions with GenAI-based VPs.

**Methods:**

Second-year medical students (N=210) participated in 5 history-taking cases with GenAI-based VPs, yielding 1030 dialogues. Researchers applied the systematic text condensation method to these dialogue data from cases 1 to 4 to inductively develop a coding scheme and validate coding consistency. Subsequently, the dialogue data from case 5 were used to assess the correlation between the students’ history-taking behaviors and their academic performance, including diagnostic accuracy, history-taking checklist scores, clinical knowledge test scores, and postencounter form scores.

**Results:**

A coding scheme comprising 12 behaviors across 3 dimensions—clinical reasoning behaviors, information gathering behaviors, and social interaction behaviors—was developed with high interrater reliability (κ≥0.85). The correlation analysis revealed that key clinical reasoning behaviors, such as *summarizing and integrating* and *logical organization*, showed significant positive correlations with multiple performance metrics, underscoring their importance in fostering clinical competence. In contrast, information gathering behaviors such as *specifying symptoms* and *routine question* were associated with clinical knowledge and thoroughness of history taking but were less predictive of diagnostic accuracy.

**Conclusions:**

This study developed a reliable, theory-informed coding scheme that can identify students’ questioning behaviors during history taking with GenAI-based VPs. The scheme effectively captures higher-order cognitive strategies and provides valuable insights into the development of clinical reasoning in medical students. This approach offers a scalable and efficient way to integrate real-time feedback into future medical education, fostering personalized learning and advancing competency-based assessments in clinical training.

## Introduction

Clinical reasoning is a core cognitive process in which clinicians gather, interpret, and analyze patient information to generate and test diagnostic hypotheses and ultimately make appropriate clinical decisions [1-3]. Clinical reasoning begins early during the initial patient encounter, as initial cues are used to generate a small set of hypotheses that guide subsequent information gathering and are iteratively tested during history taking, physical examination, and diagnostic testing [3-5]. As the first stage of clinical reasoning in patient encounters, history taking is a key step for information gathering and exerts a central influence on subsequent reasoning and decision-making [6-9]. Through patient-centered interviewing, targeted questioning, and attentive listening, effective history taking helps clinicians identify salient information, detect subtle clues, explore symptoms comprehensively, and rule out critical red-flag conditions [10,11]. Theoretically, clinicians’ questioning strategies during history taking can be explained by hypothetical-deductive reasoning [5], illness script theory [12], and dual-process models [1,13-15]. Early hypothesis generation is closely linked to diagnostic accuracy [16]. In parallel, novices frequently experience challenges in eliciting and discriminating relevant cues during history taking, underscoring the need for assessment approaches that capture clinical reasoning in this phase and enable targeted educational interventions [17,18].

Current assessment methods evaluating students’ clinical reasoning abilities in history taking have several limitations. Traditional scoring tools, such as checklist-based systems during history taking, predominantly assess students’ recall abilities rather than their active clinical reasoning processes [18,19]. Students achieve high scores mainly by remembering and mechanically checking off predetermined items, potentially inflating their performance while inadequately reflecting actual clinical reasoning skills [20]. Additionally, more advanced assessment formats, such as key feature questions, script concordance tests, and postencounter written reflections [18,21,22], attempt to indirectly measure clinical reasoning through evaluating the quantity and accuracy of collected patient information and diagnoses [23] but are limited in capturing the dynamic, ongoing cognitive process occurring during real-time patient interactions [24] and providing personalized and timely feedback to learners.

To better prepare students for real-world clinical practice, educational initiatives and assessments must move beyond rote questioning to foster and evaluate the dynamic integration of history-taking skills and clinical reasoning abilities as an interactive process [7,25]. Innovative and practical methods are required to directly capture how students’ history-taking strategies interact with their clinical reasoning skills [26,27]. Hasnain et al [28] classified history-taking strategies by analyzing how frequently students inquired about key symptoms and how thoroughly they explored presenting complaints. However, such surface-level metrics, although useful for identifying hypothesis-driven inquiry [29], fail to capture the deeper cognitive strategies central to clinical reasoning [8]. Haring et al [30] identified observable indicators of clinical reasoning across 4 domains—student actions, patient responses, conversational flow, and data gathering efficiency—through expert evaluation of students’ video-recorded performances. Both Hasnain et al [28] and Haring et al [30] relied primarily on expert subjective judgment, reflecting a lack of clearly operationalized behavioral definitions. Although this approach effectively captures the interactive nature of clinical reasoning, it depends heavily on expert input and labor-intensive video analysis, limiting its scalability and consistency [31]. Operationalizing the qualitative framework proposed by Haring et al [31], Fürstenberg et al [32] developed the Clinical Reasoning Indicators-History Taking-Scale (CRI-HT-S), a structured assessment scale encompassing domains such as “focusing questions,” “creating context,” and “securing information.” Despite these refinements, the CRI-HT-S still relies on subjective evaluation and intensive rater training; demonstrates only moderate internal consistency [33]; and is limited in capturing the depth and nuance of students’ reasoning, as shown in large-scale implementations [34]. Taken together, these studies reveal a critical gap: the limited ability to conduct standardized analyses using extractable dialogue data.

The growing adoption of virtual patient (VP) simulations, including conversational agents and generative artificial intelligence (GenAI)–based VPs, has created interactive, low-risk environments in which students can repeatedly practice and refine clinical reasoning skills in authentic clinical scenarios [35-38]. In addition to supporting experiential learning, these platforms generate structured conversational data, offering unique opportunities for real-time analysis of reasoning behaviors. In response to these opportunities and the limitations of existing assessment methods, this study aimed to develop and validate a novel coding scheme tailored to capture and assess medical students’ clinical reasoning processes and history-taking competence during consultation dialogues with a GenAI-based VP. Such a scheme could offer the potential to enable real-time, large-scale tracking of learners’ evolving clinical reasoning skills, translating spontaneous conversational behaviors into direct, interpretable, and reliable indicators of proficiency. By operationalizing clinical reasoning in this way, the proposed scheme could support both individualized feedback and rigorous, scalable assessment in technology-enhanced medical education. To evaluate the effectiveness of this approach, this study addresses the following research question: to what extent can a coding scheme reliably and validly capture medical students’ clinical reasoning competence during consultation dialogues with a GenAI-based VP?

## Methods

### Participants

Participants were second-year medical students at a university medical college in Shantou, China, enrolled in their second semester (N=210). At the time of data collection (April to May 2024), students had completed learning about systemic anatomy and were studying preclinical medicine, including cardiovascular and respiratory sciences. During this period, students began to construct their foundational illness scripts and develop basic skills in history taking and symptom recognition. Because participants had not yet received formal training in complex diagnostic reasoning, we selected common chest pain presentations aligned with their curriculum to reduce construct-irrelevant variance due to advanced specialty knowledge.

### Ethical Considerations

This study was approved by the Ethical Committee of Medical College, Shantou University, China (SUMC-2024‐064). All participants received both oral and written information regarding the study’s objectives and the voluntary nature of their participation. Written informed consent was obtained from all participants prior to data collection, with the explicit right to withdraw at any time without penalty, whereupon their data would be deleted and excluded from analysis. To ensure privacy and confidentiality, all collected data were anonymized and coded for analysis. Identifiable information was stored on secure, password-protected systems accessible only to the research team. Participants received a financial compensation of ¥20 (US $2.9) for each clinical case completed.

### Data Collection Design

Students engaged in history-taking and clinical reasoning exercises with a GenAI-based VP operationalized as a chatbot [39]. The chatbot used a tailored prompt designed for GPT-3.5, provided in Multimedia Appendix 1. The chatbot was integrated in FLoRA [40,41] to facilitate these interactions. FLoRA is a Moodle-integrated digital learning platform specifically designed to record the entirety of each student-VP dialogue for comprehensive subsequent analysis. The study incorporated 5 representative “chest pain” cases—spontaneous pneumothorax, stable angina, aortic dissection, acute pulmonary embolism, and acute pericarditis—selected for their clinical relevance and diversity in diagnostic challenges. Across all 5 cases, a total of 1030 consultation dialogues were collected, with each case involving between 205 and 207 of the 210 participants (Table 1). For this study, we defined a consultation dialogue as an exchange between a medical student and a VP. A dialogue turn referred to 1 question-answer pair between the student and the VP. Case 1 yielded a higher average number of dialogue turns compared to subsequent cases. This increase in dialogue turns can be attributed to students’ initial tendency to ask redundant questions and engage in exploratory interactions while acclimating to the FLoRA system.

**Table 1.** Dialogue turn statistics collected from 5 cases in the study.

Case	Participants, n	Total dialogue turns, n	Dialogue turns per participant, mean (SD)
1	206	18,792	91.2 (31.2)
2	207	14,753	71.3 (13.7)
3	205	15,379	75.0 (11.8)
4	206	15,723	76.3 (13.3)
5	206	15,911	77.2 (16.8)

### Coding Scheme Development

The development of the coding scheme followed a rigorous, iterative process that integrated deductive theoretical grounding with inductive semantic analysis.

#### Deductive Foundation

The deductive coding framework was informed by prior empirical studies that examined observable behaviors during medical history taking and their relationship to clinical reasoning. In particular, we drew on the behavioral classifications described by Hasnain et al [28] and Haring et al [30], as both studies explicitly linked observable student actions during history taking to indicators of diagnostic reasoning. Hasnain et al [28] identified specific positive and negative history-taking behaviors associated with diagnostic competence, providing a structured set of behavioral indicators. Haring et al [30] used expert observation and qualitative analysis of video-recorded encounters to identify moments where clinical reasoning was demonstrated or absent in student performance. These frameworks were used as conceptual starting points for developing the initial coding scheme. The behavioral categories were subsequently adapted and operationalized to fit the context of student interactions with GenAI-based VPs. Rather than adopting them verbatim, we translated each construct into explicit, dialogue-level linguistic and interactional indicators and developed decision rules to ensure consistent coding in the interactions with GenAI-based VPs. Where necessary, we refined and split constructs to increase analytic precision (eg, separating summarizing-as-integration from summarizing-as-restatement; Multimedia Appendix 2).

#### Inductive Refinement

To ensure that the scheme captured the nuances of student-VP interactions, we further refined these categories inductively using Malterud’s [42] systematic text condensation (STC). In the study, “meaning units” referred to students’ individual utterances during interactions with GenAI-based VPs. Each utterance was treated as a discrete unit for analysis, with 1 or more codes assigned as needed on the basis of the specific intent of the inquiry. In defining each code, we drew on the principles of discourse and content analysis [43], framing the codes around 3 aspects: linguistic form, pragmatic intent, and contextual function. When inductive findings extended beyond or diverged from the initial deductive categories, we expanded the conceptual scope of existing constructs, differentiated them into analytically distinct subcategories, or introduced new codes to ensure comprehensive capture of observed behaviors. This process resulted in a theory-informed framework refined and supplemented through systematic semantic analysis. The evolution from these theoretical indicators to the finalized codes, detailed through the 4-step STC, is presented in Table 2.

**Table 2.** Coding scheme development process based on systematic text condensation.

Step	Focus (Malterud’s terminology)	Description
1	Total impression—from chaos to themes	NC reviewed all consultation dialogues (cases 1‐4) to gain a holistic understanding. Through a combination of deductive mapping from established theory [28,30] and initial inductive semantic analysis, we identified core themes such as symptom inquiry, pathophysiological thinking, and other emergent interaction behaviors, forming a preliminary thematic scheme.
2	Identification and sorting of meaning units—from themes to codes	Dialogues from case 1 were analyzed to identify “meaning units.” These units were sorted and compared against the initial themes. Through this inductive process, redundant codes were removed and similar codes merged; for example, *summarizing* was refined into 2 distinct codes based on semantic synthesis levels—*summarizing and integrating* and *summarizing and restating*.
3	Condensation—from code to meaning	The refined scheme was applied to dialogue data from cases 2‐4 to ensure robustness across diverse clinical contexts. Through cross-case comparison, we condensed meaning units into validated “condensates,” further clarifying the boundaries and definitions of each code to ensure consistency in the analysis of student behaviors.
4	Synthesis—from condensation to descriptions and concepts	The final condensates were synthesized into 12 formalized codes across 3 dimensions. This step involved a final conceptual refinement to ensure that the scheme accurately reflected clinical reasoning constructs. The resulting scheme was finalized as a standardized tool applicable to varied student-VP^a^ interaction scenarios.

a VP: virtual patient.

#### Coding Scheme Refinement

To further ensure reliability, 2 authors (NC and YL), with backgrounds in clinical practice and medical education, independently conducted an initial thematic analysis of the case 1 dialogue data and collaboratively refined the coding scheme through iterative discussions. Cases 1 to 4 served as the iterative development and refinement dataset during the construction of the coding scheme. Then, NC and YL randomly selected 20 students and coded their dialogue data from cases 1 to 4. Discrepancies were resolved through iterative discussions to refine code definitions and boundaries. This evolution process, including the merging of redundant codes and the refinement of meaning units, is detailed in Multimedia Appendix 2.

For interrater reliability calibration, subsets of dialogue turns were randomly sampled and independently coded. Cohen κ was calculated on prediscussion ratings, and the calibration was conducted in multiple iterative rounds with progressively refined code definitions and decision rules until all codes achieved the predefined reliability threshold (κ≥0.85). A detailed account of the multiround reliability assessment procedure is provided in the *Results* section. Once the coding scheme was finalized, all case dialogues were fully coded, with 2 authors (NC and YL) each independently coding half of the dataset.

### Applying and Validating the Coding Scheme

The coding scheme was applied to the entire corpus of 80,558 dialogue turns collected from 210 students over the 5-week study period. All dialogue data were manually coded by researchers, ensuring that the coding scheme captured the full volume of dialogues. For a detailed breakdown of all code frequencies and their distribution from case 1 to case 5, please see Multimedia Appendix 2.

To evaluate the coding scheme in a phase distinct from its iterative development, we conducted correlation analyses using case 5 (acute pericarditis). Cases 1 to 4 were used for coding scheme development and refinement; thus, using case 5 for this analysis reduced the potential bias between scheme construction and validation. Moreover, case 5 was the most diagnostically complex case in the sequence, providing a context in which clinical reasoning behaviors could be examined under higher complexity and stability. By coding these dialogues, we were able to systematically compare student behaviors as captured by the coding scheme against widely accepted criteria for assessing clinical reasoning and history-taking skills. Specifically, the coded data were correlated with multiple external benchmarks validated in prior research, allowing us to examine whether code frequencies were associated with meaningful aspects of clinical reasoning performance. These included students’ clinical knowledge test scores; diagnostic accuracy, defined dichotomously as correct or incorrect; history-taking checklist scores, adapted from the Kalamazoo Essential Elements Communication Checklist and Medical Licensing Examination criteria; and scores from a modified postencounter form [20,44-47]. These measures provided a comprehensive context for validating the relevance and sensitivity of the coding scheme to key aspects of student performance.

To quantitatively assess the relationships between code frequencies and established assessment criteria (referred to as concurrent validity) [48], we conducted Pearson correlation analysis for continuous codes and point-biserial correlation analysis or chi-square tests for binary codes. These analyses were based on the counts of codes per participant, the frequency of individual code occurrence, and students’ scores on all other assessments described above. All statistical analyses were performed in Python (Python Software Foundation), with a significance threshold of *P*<.05. To control for multiple comparisons, we applied the Benjamini-Hochberg method for *P* value correction. By triangulating findings from various statistical tests across multiple performance metrics, this validation process provided empirical support for the theoretical coherence and practical applicability of the final coding scheme within the context of VP-based clinical reasoning education.

## Results

### Final Coding Scheme

The finalized coding scheme comprised 12 behavioral codes aimed at capturing students’ clinical reasoning, information gathering, and social interaction behaviors during history taking (see Textbox 1 for an overview of these dimensions and their related codes). All student utterances were assigned at least 1 code across the 3 behavioral dimensions, ensuring comprehensive coverage of the dialogue corpus. A complete example of student-patient dialogue coding is provided in Multimedia Appendix 3.

Textbox 1. **Dimensions and codes for identifying students' history-taking behaviors.**

**Dimension 1: clinical reasoning behaviors**

- Pathophysiological question
- Relevant response
- Summarizing and integrating
- Logical organization

**Dimension 2: information gathering behaviors**

- Specifying symptoms
- Routine question
- Summarizing and restating
- Checking
- Repeating question
- Fuzzy question

**Dimension 3: social interaction behaviors**

- Facilitative communication
- Off-topic statement

### Detailed Explanation of Codes, Definitions, and Examples

#### Dimension 1: Clinical Reasoning Behaviors

This dimension referred to the cognitive synthesis of patient data to generate targeted, hypothesis-driven inquiries. It focused on how students analyzed the obtained information to formulate purposeful questions that test or refine clinical diagnostic possibilities.

##### Pathophysiological Question

When students posed specific, hypothesis-driven questions grounded in pathophysiological thinking—such as asking about radiating pain at anatomically relevant sites, identifiable triggers or alleviating factors, or relevant past or family history—these behaviors were coded as *pathophysiological question*. The code also applied when students provided diagnostic explanations or suggestions in response to patient concerns. The defining feature of this behavior is its clear diagnostic intent. For example, in a case involving stable angina, the following responses of students were coded as *pathophysiological question*:

*Okay, have you had any heart problems before?*

*The preliminary diagnosis is angina, but I'll confirm it after you have an ECG.*

*Did the pain always occur when you were lifting heavy objects or climbing slopes?*

##### Relevant Response

When patients mentioned diagnostically significant information such as characteristic symptoms, key clinical signs, or high-risk factors, the responses of students who recognized and responded to these features were coded as *relevant response*. The defining feature of this behavior is the student’s ability to pick up on clinical cues provided by the patient and follow up with targeted inquiries. For example, the following student responses were coded as *relevant response*:

*For how long have you been taking them? [in case of pulmonary embolism, responding to oral contraceptive use]*

*How long after taking the medication does the pain relief occur? [in case of stable angina, responding to nitroglycerin use]*

##### Summarizing and Integrating

Summarizing and integrating the patient’s information in a coherent and structured manner was coded as *summarizing and integrating*, a binary code. This behavior was not limited to repetition but involved logical reorganization of collected data, as illustrated in the following example:

*You experienced sudden, persistent pain in your right chest this morning after forcefully blowing up a balloon, which lasted about 2 minutes before gradually easing, but it hasn’t been completely relieved even after resting. You’ve had bilateral chest tightness that persists until now, and it worsens noticeably when walking quickly or climbing stairs....*

##### Logical Organization

This code was assigned on the basis of the diagnostic logic underlying a question rather than its sequential order. It identified the exploration of pertinent positive or negative associated symptoms clinically relevant to the chief complaint. Specifically, while *pathophysiological question* evaluated the mechanistic depth of an inquiry, *logical organization* captured the student’s strategic ability to link the primary complaint with other clinical signs to rule in or rule out potential diagnoses, as illustrated in the following examples:

*Do you have hematuria/reduced urine output? [in case of aortic dissection]*

*Do you have shortness of breath/cyanosis? [in case of pulmonary embolism]*

### Dimension 2: Information Gathering Behaviors

This dimension encompassed the student’s adherence to standardized history-taking protocols and the application of foundational interviewing techniques. It focused on the procedural completeness of data collection as prescribed by the medical curriculum, ensuring a systematic acquisition of patient information.

#### Specifying Symptoms

This code included questions aimed at systematically exploring the characteristics of the symptom. Typical aspects included onset, duration, location, quality, severity, and aggravating or relieving factors. Unlike *logical organization*, which evaluated the diagnostic logic used to link multiple symptoms, *specifying symptoms* was strictly confined to the descriptive characterization of a single symptom. Furthermore, while *pathophysiological question* explored underlying pathophysiological mechanisms, *specifying symptoms* focused on the external attributes of the clinical presentation. Some examples are presented below:

*What do you think triggered your chest pain today?*

*Are there any factors that worsen or relieve the pain?*

#### Routine Question

*Routine question* referred to items commonly asked in all clinical interviews, regardless of the specific presenting problem. These included standard elements such as basic patient information, past medical history, personal and family history, and review of systems—questions that resembled those found on clinical interview checklists. This category also included commonly used open-ended questions in routine history taking.

*What discomfort brought you to the clinic?*

*Do you have any other discomfort that occurred at the same time as this chest pain?*

*How old is your father?*

#### Summarizing and Restating

This code was applied when students summarized collected information by restating it without reorganization or synthesis. Unlike *summarizing and integrating*, which required thematic integration of data, *summarizing and restating* was characterized by a “linear playback” of facts, typically in chronological order, where the summary lacked a diagnostic or structured approach to reformulation. *Summarizing and restating* and *summarizing and integrating* are binary and mutually exclusive; any summarizing action must be categorized as one or the other but not both. An example of *summarizing and restating* is given below:

*Two minutes ago, you came to the hospital due to chest pain and shortness of breath. You haven’t sought medical attention before, nor have you taken any medication....*

#### Checking

This code was applied when students sought to confirm or clarify patient-reported information, typically in response to unclear or seemingly inconsistent statements. *Checking* was often used in combination with other codes, such as *routine question*, *specifying symptoms*, or *logical organization*, when confirmation occurred alongside other history-taking approaches. An example of *checking* behavior is shown below:

***Patient:** It lasted for about 2 minutes.*

***Student:** So, after 2 minutes, the pain became the same as it is now?*

#### Repeating Question

*Repeating question* was characterized by students asking about the same clinical information point multiple times, sometimes using different expressions or synonymous terms. However, whether such synonymous expressions would be coded as *repeating question* depended on the student’s level of clinical knowledge. Among lower-year medical students, this behavior is often associated with limited understanding of clinical terminology. For instance, they may treat synonymous terms—such as “shortness of breath” and “labored breathing”—as distinct symptoms. Consequently, such instances may result in alternative coding. The following example illustrates a *repeating question* behavior where the semantic intent remained redundant despite the change in phrasing:

***Student:** Do you experience shortness of breath or chest tightness? [logical organization]*

***Patient:** I feel my heart racing, sweating, and lightheaded, but without shortness of breath or chest tightness.*

*[....17 rounds later]*

***Student:** Is your breathing normal when you have chest pain? [repeating question]*

#### Fuzzy Question

*Fuzzy question* involved the repeated use (2 or more times) of vague, open-ended questions within a single section of history taking, typically intended to prompt patients to provide additional information voluntarily. This definition excluded appropriate opening inquiries, which were coded as *routine question*. An example illustrating the distinction between *fuzzy question* and *routine question* is provided below:

***Student:** How was your health before? [routine question]*

*....*

***Student:** Do you have any other medical conditions besides high blood pressure? [routine question]*

*....*

***Student:** Any other diseases? [fuzzy question]*

### Dimension 3: Social Interaction Behaviors

This dimension involved facilitative verbal interactions that serve to maintain rapport and ensure a smooth conversational flow. It focused on empathetic expressions, active listening cues, and other supportive utterances that foster the patient-interviewer relationship rather than direct clinical data collection.

#### Facilitative Communication

*Facilitative communication* included greetings, small talk, brief responses, transitional phrases, terminology explanations, expressions of reassurance, and nondiagnostic patient education. When brief conversational elements (eg, “Okay”) appeared at the beginning of a question, they were not independently coded as *facilitative communication*. Instead, the entire utterance was coded according to the category assigned to the question itself (eg, *fuzzy question*, *routine question*, *logical organization*, and *specifying symptoms*), in order to avoid overcoding. However, if they were semantically distinct from other coded behaviors, dual coding was applied, assigning both *facilitative communication* and the relevant category. The following examples illustrate independent *facilitative communication* behaviors:

*Hello, I'm Dr. Guo. [greeting]*

*Take it easy. [reassurance]*

*Hemoptysis means you coughed up and noticed blood. [terminology explanation]*

#### Off-Topic Statement

Utterances that were incomplete, illogical, or entirely unrelated to the clinical case and therefore could not be reasonably classified under any other code were categorized as *off-topic statement* to ensure coding integrity and comprehensive inclusion of all dialogue segments:

*What kind of bird do you keep, pigeons?*

*Was the balloon sucked in?*

### Coding Combinations

To capture the complexity of student-patient interactions, multiple codes were assigned to a single utterance whenever it encompassed more than one functional meaning unit. This approach was not restricted to predefined combinations but was driven by the specific intent of the inquiry. This granular coding allowed for a more nuanced assessment of how students navigated between routine inquiry, symptom exploration, and diagnostic reasoning. A comprehensive taxonomy of these combinations and illustrative examples are provided in Multimedia Appendix 3.

### Interrater Reliability of the Coding Scheme

Interrater reliability was established through a 4-round calibration of the 12-code scheme. In each round, we randomly sampled student utterances (≤100 utterances per student) and ensured that κ estimation for each code under evaluation was based on at least 80 independently labeled utterances [49]. Round 1 included 1034 utterances from 13 students; because several codes occurred too infrequently to support separate κ estimation, we assessed reliability at a higher level by pooling *pathophysiological question*, *relevant response*, and *summarizing and integrating* into 1 category for κ computation (κ=1.00), pooling *summarizing and restating*, *checking*, *repeating question*, and *fuzzy question* into another category for κ computation (κ=0.96) and pooling *facilitative communication* and *off-topic statement* into a group for κ computation (κ=1). For the higher-frequency individual codes, strong agreement was likewise observed: *logical organization* (κ=1.00, 95% CI 1.00-1.00), *routine question* (κ=0.99; 95% CI 0.96-1.00), and *specifying symptoms* (κ=0.96; 95% CI 0.89-1.00). After each round, 2 raters independently coded the sampled utterances, and Cohen κ was computed on the prediscussion ratings. Codes (or pooled code groups) meeting the criterion (κ≥0.85) were removed from subsequent rounds, and remaining disagreements were reviewed to refine code definitions and decision rules. Rounds 2, 3, and 4 sampled 600 utterances from 8 students, 200 utterances from 2 students, and 100 utterances from 2 students, respectively, again maintaining ≥80 labeled utterances per remaining code (or pooled group). This process continued until all codes (or pooled code groups) achieved κ ≥0.85. We report κ with 95% confidence intervals estimated via bootstrap resampling (10,000 resamples).

### Associations Between Codes and Performance—Validity

The coding scheme demonstrated strong construct validity, as evidenced by significant correlations between specific behaviors and performance metrics (Table 3). Table S1 in Multimedia Appendix 2 shows the basic statistical distribution (minimum, maximum, mean, and SD) of student behavior codes and performance metrics. This provides an overview of the data’s range and variability. Advanced clinical reasoning behaviors—specifically *summarizing and integrating* and *logical organization*—emerged as the primary differentiators of clinical proficiency. *Summarizing and integrating* was the most robust predictor, showing consistent, significant correlations across all measures, including diagnostic accuracy (*P*=.048). Similarly, *logical organization* served as a key indicator of success. It highlights that maintaining internal coherence by linking chief complaints with logically related symptoms is vital for an accurate diagnosis.

**Table 3.** Correlation between behavior codes and performance metrics in case 5 (acute pericarditis).^a^

Code	Diagnostic accuracy		History-taking checklist score		Clinical knowledge test score		Postencounter form score	
	*r*/*(χ²)*	*P* value	*r*	*P* value	*r*	*P* value	*r*	*P* value
Dimension 1: clinical reasoning behaviors								
Pathophysiological question	0.082	.619	0.187	.019	0.154	.084	0.119	.273
Relevant response	−0.062	.653	0.082	.418	−0.014	.914	−0.025	.871
Summarizing and integrating	8.389	.048	0.252	<.001	0.236	.006	0.239	.012
Logical organization	0.186	.048	0.570	<.001	0.178	.044	0.144	.164
Dimension 2: information gathering behaviors								
Specifying symptoms	0.030	.730	0.344	<.001	0.262	<.001	0.067	.643
Routine question	0.141	.184	0.380	<.001	0.032	.873	0.188	.042
Summarizing and restating	0.190	.730	−0.071	.447	−0.012	.914	0.050	.643
Checking	−0.044	.715	0.019	.790	−0.129	.134	0.010	.889
Repeating question	0.049	.715	0.068	.447	−0.108	.214	0.051	.643
Fuzzy question	−0.070	.650	−0.057	.456	0.032	.873	−0.062	.643
Dimension 3: social interaction behaviors								
Facilitative communication	−0.006	.929	0.106	.268	0.141	.108	0.017	.878
Off-topic statement	0.080	.619	0.060	.456	0.008	.914	0.053	.643

a Pearson correlation analysis was used for continuous codes, while point-biserial correlation or *χ2* test was used for binary codes (*summarizing and integrating* and *summarizing and restating*) and binary performance (diagnostic accuracy). The *P* value was corrected with the Benjamini-Hochberg method.

In contrast, while foundational behaviors such as *specifying symptoms* and *routine question* contributed to the thoroughness of history taking, they were less predictive of diagnostic accuracy. Notably, *specifying symptoms* also showed a strong correlation with clinical knowledge scores (*P*<.001), suggesting that detailed symptom exploration is closely linked to a student’s underlying knowledge base. Beyond these primary predictors, *pathophysiological question* was associated with higher thoroughness of history taking (*P*=.019), while *routine question* showed a narrow correlation with the postencounter form score (*P*=.042).

These findings suggest a clear hierarchy: while basic inquiry and data collection form the bulk of the encounter, clinical excellence is defined by high-level synthesis and the internal logical coherence of the inquiry process.

## Discussion

### Principal Findings

This study developed an operationalized, dialogue-based coding scheme for capturing medical students’ behaviors during history taking with a GenAI-based VP. Building upon deductive blueprints, we used STC-informed iterative refinement to translate theoretical constructs into 12 observable behaviors. These behaviors, spanning clinical reasoning, information gathering, and social interaction, demonstrated high interrater agreement after structured calibration. We further provided validity evidence based on correlations with other variables: behaviors reflecting higher-order synthesis and diagnostic organization (eg, *summarizing and integrating* and *logical organization*) were more consistently associated with performance outcomes than routine or socially facilitative utterances.

### Differentiating Deep Reasoning From Surface-Level Behaviors

The positive associations of *summarizing and integrating* and *logical organization* with multiple performance indicators are consistent with prior work linking diagnostic expertise to structured synthesis and clinically meaningful organization of information [22,50,51]. *Summarizing and integrating* reflects learners’ ability to integrate and reorganize clinical data into a coherent representation. *Logical organization* shifts the focus from the sequence of questions to their clinical relevance, thereby operationalizing diagnostic organization more directly than order-based approaches [30].

In contrast, *pathophysiological question* was infrequent and only weakly related to the thoroughness of history taking, which likely reflects participants’ early stage of biomedical knowledge and limited capacity to translate mechanistic concepts into effective hypothesis testing in real time [52,53]. *Relevant response* also showed no clear associations with performance, suggesting that novice learners may respond to salient cues reactively rather than strategically, consistent with underdeveloped illness scripts and hypothesis formulation skills [15,54,55].

Although *routine question* and *specifying symptoms* were common, their weaker links to performance suggest a “wide-net” approach emphasizing checklist completeness over interpretive reasoning [18,19]. Similarly, *checking*, *fuzzy question*, and *repeating question* may reflect attempts to manage uncertainty or cognitive load rather than deliberate diagnostic strategy [56]. *Facilitative communication* and *off-topic statement* primarily serve social or conversational functions, with no relevance to clinical problem-solving [57].

Overall, the pattern of associations supports the scheme’s sensitivity to the semantic quality of inquiry: it distinguishes higher-order, logically connected reasoning moves from procedural data collection and nonclinical talk and thus provides a plausible foundation for future automated assessment and feedback.

### Comparative Advantages and Generalizability of Process-Oriented Assessment

Building on prior work [28,30], we extend existing descriptions of history taking by introducing additional, operationalized behavioral indicators organized into 3 dimensions. The scheme is designed for AI-mediated learning environments: its explicit definitions support reliable human coding and provide a structured target for future automated classification and feedback. Compared with established clinical reasoning assessments, this dialogue-based approach offers complementary advantages for capturing reasoning in action. Instruments such as script concordance tests [21], clinical reasoning problems [58], and postencounter forms [18] benchmark learners’ judgments against expert responses but provide limited visibility into how questions are selected and adapted during an encounter [18,58]. Oral examinations can elicit reasoning through conversation but are susceptible to examiner effects and variable reliability [59]. Objective structured clinical examinations (OSCEs) and key feature questions, while widely used, often prioritize checklist completeness or discrete decision points and may not represent the continuity of reasoning across an interview [20,60]. In contrast, our scheme codes the pragmatic intent of student utterances, enabling process-oriented assessment from naturalistic learner-patient dialogues.

Recent GenAI-enabled training systems highlight the feasibility of scalable simulation and feedback (eg, Brügge et al [34]) but commonly rely on existing inventories rather than modeling fine-grained dialogue behaviors. Our scheme addresses this gap by providing an interpretable representation of reasoning-related dialogue moves that can complement GenAI-based simulation and feedback.

Finally, the stability of code distributions across cases (Table S2 in Multimedia Appendix 2) suggests that the scheme may capture generalizable reasoning patterns beyond a single case. Ongoing work is extending the coding scheme to additional presenting complaints (eg, fever, dyspnea, and abdominal pain) to further test generalizability and reliability.

### Educational Implications

The findings of this study have significant implications for the evolution of digital medical education in the age of AI.

First, the scheme provides an interpretable “intermediate representation” of learners’ dialogue moves that is amenable to automation. Because the codes are defined in terms of pragmatic intent (eg, hypothesis testing vs descriptive symptom elicitation) rather than case-specific keywords, they can serve as targets for supervised natural language processing classifiers and GenAI-based annotators [61,62]. Importantly, any move toward automated coding should be framed as an empirical program rather than an assumption: future work should report classification performance (eg, macro-F1 for imbalanced codes) [63], robustness across cases and model versions, and calibration for high-stakes use before deployment in assessment contexts.

Second, a dialogue-level behavioral profile can enable more actionable formative feedback than checklist completeness alone. Code patterns such as frequent *routine question* responses without evidence of synthesis, limited *logical organization*, or failure to respond to diagnostically salient cues can be translated into specific, teachable feedback messages and targeted prompts during subsequent practice sessions [64]. For example, learners who rarely demonstrate *logical organization* could receive scaffolds that explicitly cue key discriminating positives or negatives for competing diagnoses, whereas learners who do not produce *summarizing and integrating* statements could be prompted to generate a 1- to 2-sentence problem representation before advancing to new topic areas. Such feedback is potentially scalable, but it should be evaluated for educational impact (eg, improvement in subsequent dialogue behaviors and diagnostic accuracy) and for unintended consequences (eg, gaming the codes or reduced attention to rapport) [65].

Finally, the scheme supports longitudinal, process-oriented assessment of developing reasoning. By tracking observable reasoning behaviors across repeated encounters, educators can complement outcome measures (eg, diagnostic accuracy) with indicators of the reasoning process and progression, enabling growth-oriented competence monitoring over time [66]. Because the codes capture generalizable discourse functions rather than disease-specific scripts, the approach may support transfer across clinical domains and stages of training; nevertheless, claims about lifelong applicability require validation across learner levels, institutions, and authentic clinical contexts [67]. Overall, the scheme offers a structured bridge between history-taking instruction and data-driven, feedback-oriented educational systems while highlighting the need for rigorous evaluation of automation and downstream educational consequences.

### Limitations and Future Directions

Several limitations warrant consideration. First, the study was conducted at a single institution and included only second-year students, which may limit generalizability. Second, although interrater reliability was high, manual coding limited its scalability in real-time settings. Third, while this study used 5 chest pain cases to ensure a controlled baseline appropriate for second-year students’ cognitive stage, the current scope remains a limitation regarding generalizability. However, its focus on underlying cognitive mechanisms, rather than on disease-specific keywords, suggests potential utility in other domains. Our ongoing follow-up study involving 8 additional cases (eg, abdominal pain and fever) will further validate this cross-domain robustness and build upon the coding stability observed in the preliminary analysis.

Future work should explore the automation of the coding process using GenAI to enable real-time assessment and feedback. Longitudinal studies tracking students’ reasoning development over time and across clinical encounters would help validate the scheme’s utility. Further integration with formative assessments, OSCEs, or AI-driven learning platforms could support its transition from a research tool to an embedded component of clinical education at scale.

### Conclusions

We developed an operationalized 12-code scheme to identify students’ clinical reasoning–relevant behaviors during history taking with GenAI-based VPs. The scheme demonstrated high interrater agreement and provided validity evidence based on correlations with other variables, with synthesis and diagnostic organization behaviors showing stronger alignment with performance outcomes than procedural behaviors. This work establishes a reproducible foundation for process-oriented assessment of history taking and supports future research on automated coding and feedback across broader clinical domains and learner populations.

## Supplementary material

10.2196/84347 Multimedia Appendix 1 Prompt template for generative artificial intelligence–based virtual patients.

10.2196/84347 Multimedia Appendix 2 Detailed development of the coding scheme, correlation with performance metrics, and cross-case distribution.

10.2196/84347 Multimedia Appendix 3 Sample of student–virtual patient dialogue with codes.

## Abbreviations

AI: artificial intelligence
CRI-HT-S: Clinical Reasoning Indicators-History Taking-Scale
GenAI: generative artificial intelligence
OSCE: objective structured clinical examination
STC: systematic text condensation
VP: virtual patient
