# Supplementary material for: Developing and Validating a Coding Scheme for Clinical Reasoning in History Taking Using Generative AI–Based Virtual Patients: Systematic Text Condensation Approach
Source: JMIR Med Educ. 2026 Apr 13;12:e84347. doi: 10.2196/84347 (PMC13075466; doi:10.2196/84347)
Supplement: Multimedia Appendix 2 [file mededu-v12-e84347-s002.docx]

**Appendix 2. Detailed Development of the Coding Scheme, Cross-Case Distribution, and Correlation with Performance Metrics**

## Iterative Refinement and Conceptual Definition of Codes

### Refinement of Conceptual Definitions

**Specifying symptoms**

Initially, this code referred to inquiries focusing on the basic characteristics of chest pain, such as triggers, duration, time of first occurrence, location, and severity. Example:

*"What may cause your chest pain?"*

Subsequently, we expanded the code definition to include more specific elements, such as confirmation of the initial occurrence time and changes in severity. Examples:

*"Is this the first time you've had chest pain?",*

*"Will the chest pain get worse?"*

Finally, the code was broadened to include not only general inquiries about the characteristics of chest pain but also questions about the specific manifestations of any particular symptom or issue, reflecting a more comprehensive understanding of symptom specification in clinical reasoning. This pattern is commonly observed in composite codes, as demonstrated in the examples presented below.

**Pathophysiologic Question**

Raise Specific Questions or Descriptions that point to pathophysiologic thinking. Initially, the code focused on questions that explored the etiology and pathogenesis of the disease in depth, as well as interpretive statements involving diagnostic reasoning, such as formulating preliminary diagnoses, proposing treatment suggestions, or commenting on prognosis and prevention. Example:

*"Do you often sit for long periods?" (in the context of suspected pulmonary embolism); "My initial diagnosis might be pneumothorax or an infection. I think you may need a chest CT next."*

Subsequently, the definition was expanded to include: Probing into changes in triggering factors; Exploring the relationship between associated symptoms and the chief complaint, often with consideration of underlying causes—a pattern commonly observed in composite codes; Investigating relevant past medical history that may inform current diagnostic hypotheses. Example:

*"Are all four episodes of pain the same?" "Have you had any heart problems in the past?"*

Later iterations further incorporated inquiries into the impact of a specific manifestation on the chief complaint, Example:

*"Does lying on your left side reduce the pain?"*

Finally, the code was broadened to include questions that emerge from integrated clinical reasoning, such as identifying new associated symptoms that had not been previously mentioned but are clinically relevant based on the evolving diagnostic hypothesis. Example:

*After synthesizing information from the interview, the student adds: "Let me ask one more question—have you had any shortness of breath since the onset?"*

**Logical Organization**

Initially, this code referred to inquiries about associated symptoms that were either positive findings or negative findings with differential diagnostic value.

Note: Associated symptoms without differential diagnostic value—or in-depth follow-up questions on such non-discriminative symptoms—should be coded as Routine Question. Example:

*In a pneumothorax case, the question "Do you have a cough or sputum?" was coded as Logical Organization, while "Besides chest pain, do you have any other discomfort?" was coded as Routine Question.*

Subsequently, inquiries were extended to include factors influencing associated symptoms relevant to the case, as well as questions exploring the relationship between a general symptom and the chief complaint. Example:

*"Does a change in posture affect your chest pain?"*

In composite coding, *Logical Organization* was used in conjunction with other codes when multiple elements were present. For example: When a meaningful associated symptom was explored in depth, the utterance was coded as Logical Organization+ Specifying symptoms; When symptom probing was combined with temporal clarification or confirmation, Logical Organization + Specifying symptoms + Checking was applied. Example:

*"Do you have a cough?" (Logical Organization)*

*"When did the cough first start?" (Logical Organization+Specifying symptoms )*

*Patient: "After the chest pain."*

*Student: "So it started this morning, right?" (Logical Organization+Specifying symptoms +Checking)*

**Relevant Response**

Recognizing and Responding to Relevant Information. The code was applied when a hallmark feature of the case is mentioned, the student follows up with additional questioning or confirmation regarding that specific feature. Each case in the study is characterized by the following key features.

Pneumothorax: worsening with deep breathing, balloon inflation, or coughing

Angina: onset during exertion (e.g., climbing stairs, lifting objects), tightness relieved by rest, pain may radiate to the left side of the neck; typically relieved by nitroglycerin.

Aortic dissection: tearing pain with unrelieved persistence

Pulmonary embolism: dyspnea after long-distance travel

Pericarditis: relief when sitting forward

*Example: "So the chest pain started right after you blew into the balloon? (When the patient said, "I was blowing up a balloon at the time.")*

**Summarizing & Integrating**

The code referred to the learner's effort to synthesize, structure, and verbally organize the information collected during the interview. Examples:

*"Let me summarize what we've just discussed. You had a sudden onset of persistent right-sided chest pain this morning after forcefully blowing up a balloon. It lasted about two minutes and then gradually subsided, but never fully went away. You also have bilateral chest tightness that persists and worsens with brisk walking or climbing stairs. Shortly after the chest pain, you experienced shortness of breath, which has continued until now. You had a poor appetite at noon, but your diet, sleep, mental state, and bowel/bladder habits have otherwise been normal. Did I get that right?"*

*"Okay, so when it started, you had pain in the chest and left shoulder, along with chest tightness, palpitations, sweating, and dizziness. "*

Note: When summarization occurs across two or more adjacent utterances, it should be coded only once. This rule also applies to the subsequent code, Summarizing & Restating.

**Summarizing & Restating**

The code referred to the learner's act of restating the collected information in the original order of acquisition, often as a case review or retelling. Example:

*"Let's review your condition: You came to the hospital two minutes ago because of chest pain and shortness of breath. Before that, you hadn't been to the hospital and hadn't taken any medications. Your mental state is good, your sleep is normal, your bowel and bladder functions are normal, and there's been no change in your weight. Do you think there's anything else you'd like to add?"*

**Checking**

Initially, the code was applied when the learner checked or clarified a specific detail in the patient's narrative. Example:

*"The pain gradually eased—so did it completely stop, or did it come back after you did something else?" Subsequently, the code was extended to include concise factual confirmations. Example: "The first time was half a year ago, right?"*

**Repeating Question**

Initially, this code was applied when a student repeated a question targeting the same clinical element. Examples:

*(stu_2024_ex_057)*:

*Turn 35:*

*Student: "Do you experience shortness of breath or chest tightness?" (Coded as Logical Organization)*

*Patient: "During chest pain, I felt palpitations, sweating, and dizziness, but no shortness of breath or tightness."*

*Turn 52: Student: "Was your breathing normal during the chest pain?" (Coded as Repeating Question)*

*Patient: "Yes, my breathing was normal, no dyspnea."*

Subsequently, we found that whether two questions are considered redundant depends on the learner's academic level. For example, second-year students may not recognize "shortness of breath" and "dyspnea" as referring to the same concept. In such cases, questions should be coded according to their intended clinical function rather than surface repetition. Example:

*(stu_2024_cn_013):*

*Turn 39: Student: "Have you experienced shortness of breath since the chest pain began?"*

*(Coded as Logical Organization)*

*Patient: "No."*

*Turn 50:*

*Student: "Have you had any difficulty breathing?" (Coded as Logical Organization)*

*Patient: "No, my breathing has been normal."*

### Induction of New Codes for Comprehensive Capture

The following four codes were added during the coding process, as the initial coding scheme was not sufficient to capture all types of student questions.

**Fuzzy Question**

Initially, this code was applied to all such broad questions that lacked specificity. Example:

*"Anything else?", "Any other discomfort?", or "Any other symptoms?"*

Subsequently, we observed that occasional use of open-ended questions like these is a common and acceptable clinical strategy, providing space for the patient to express concerns freely, and are often used to initiate exploration of associated symptoms. Therefore, we refined the application of this code: Fuzzy Question is assigned only when vague, open-ended questions are used for the second time or more within the same history domain. Example:

*(stu_2024_cn_002)*:

*Student: "Under what circumstances does your chest pain occur?" (Coded as Specifying symptoms)*

*Patient: "When blowing up a balloon."*

*Student: "Anything else?" (Coded as Fuzzy Question)*

*Patient: "Deep breathing."*

*Student: "Anything else?" (Coded as Fuzzy Question)*

*Patient: "It hurts when I cough."*

*Student: "Anything else?" (Coded as Fuzzy Question)*

*Patient: "It goes away after a few coughs."*

*Student: "Anything else?" (Coded as Fuzzy Question)*

*Patient: "It's better when lying on the left side."*

*Student: "Anything else?" (Coded as Fuzzy Question)*

*Patient: "No more."*

**Routine Question**

Initially, this code was applied to standard, structured inquiries commonly used in history-taking, such as demographic information, review of systems, and general condition assessment. Example:

*"What symptoms brought you to the clinic today?"*

Subsequently, the code was extended to include detailed symptom inquiries that are not directly relevant to the clinical features of the current case. If the question involves further specification of an irrelevant symptom, it may be co-coded with SS. Example:

*"May I ask about your use of nitroglycerin tablets?" (Coded as Routine Question, Specifying symptoms) — when nitroglycerin use is not pertinent to the present diagnosis.*

**Facilitative Communication**

During the coding process, this code was gradually expanded to include the following types of utterances: Greetings and self-introductions, Social niceties or polite exchanges, Non-substantive responses or verbal fillers, Transitional explanations (e.g., preparing the patient for a sensitive question), Terminology explanations, Health education, emotional reassurance, or closing remarks, These utterances serve interpersonal, procedural, or affective functions rather than diagnostic purposes. Example:

*"Hello, I'm Xiao Guo from XX Medical School." (Greeting)*

*"I'm going to ask a somewhat private question now. It might be related to your condition, so I hope you can answer honestly." (Transitional explanation)*

**Off-topic Statement**

During the coding process, this code was gradually expanded to include the following types of utterances: Clinically or logically incoherent questions, where the content does not make sense within the medical context, Repetitive questions triggered by a lack of system response (e.g., Gen-AI did not answer previously), Incomplete questions that lack sufficient clarity for interpretation or response. Example:

*"Did the balloon get sucked into your lungs?" (Clinically implausible)*

*"What kind of birds do you raise? Pigeons?" (Irrelevant or unclear context)*

*"Percussion results of the lungs." (Incomplete phrase presented as a question)*

## Association Between Behavior Codes and Performance Metrics in Case 5

Table S1 Distribution of Student Behavior Codes and Performance in Case 5 (Acute Pericarditis)

| Code / Performance | **Minimum** | **Maximum** | **Mean** | **Std. Deviation** |
| --- | --- | --- | --- | --- |
| Pathophysiologic Question | 0.00 | 12.00 | 2.50 | 2.17 |
| Relevant Response | 0.00 | 6.00 | 0.78 | 1.28 |
| Summarizing & Integrating | 0.00 | 1.00 | 0.44 | 0.50 |
| Logical Organization | 0.00 | 16.00 | 8.03 | 2.66 |
| Specifying Symptoms | 4.00 | 22.00 | 11.27 | 2.56 |
| Routine Question | 11.00 | 168.00 | 46.14 | 13.52 |
| Summarizing & Restating | 0.00 | 1.00 | 0.14 | 0.35 |
| Checking | 0.00 | 3.00 | 0.21 | 0.46 |
| Repeating Question | 0.00 | 3.00 | 0.19 | 0.52 |
| Fuzzy Question | 0.00 | 8.00 | 0.28 | 0.83 |
| Facilitative Communication | 0.00 | 15.00 | 5.13 | 2.75 |
| Off-topic Statement | 0.00 | 17.00 | 0.60 | 1.60 |
| Post-Encounter Form Score | 0.00 | 13.00 | 6.79 | 3.18 |
| History-Taking Checklist Score | 0.37 | 0.87 | 0.69 | 0.08 |
| Clinical Knowledge Test Score | 41.93 | 98.22 | 83.76 | 10.27 |
| Diagnostic Accuracy | 0.00 | 1.00 | 0.62 | 0.49 |

## Frequency and Temporal Distribution of Codes Across Clinical Cases

Table S2 Code Frequencies (F) and Percentage (%) Distribution Across the 5 cases

| Case | Case1 | | Case2 | | Case3 | | Case4 | | Case5 | |
| --- | --- | --- | --- | --- | --- | --- | --- | --- | --- | --- |
|  | F | % | F | % | F | % | F | % | F | % |
| Pathophysiologic Question | 631 | 3.38 | 356 | 2.54 | 345 | 2.41 | 533 | 3.63 | 515 | 3.30 |
| Relevant Response | 120 | 0.64 | 178 | 1.27 | 235 | 1.64 | 32 | 0.22 | 160 | 1.03 |
| Summarizing & Integrating | 70 | 0.38 | 84 | 0.60 | 71 | 0.50 | 83 | 0.57 | 92 | 0.59 |
| Logical Organization | 1611 | 8.64 | 758 | 5.40 | 1243 | 8.68 | 1672 | 11.39 | 1651 | 10.58 |
| Specifying Symptoms | 2557 | 13.71 | 2348 | 16.73 | 2188 | 15.28 | 2258 | 15.38 | 2322 | 14.89 |
| Routine Question | 11066 | 59.33 | 9154 | 65.22 | 8513 | 59.44 | 8784 | 59.84 | 9503 | 60.92 |
| Summarizing & Restating | 46 | 0.25 | 31 | 0.22 | 29 | 0.20 | 33 | 0.22 | 30 | 0.19 |
| Checking | 225 | 1.21 | 100 | 0.71 | 58 | 0.40 | 61 | 0.42 | 43 | 0.28 |
| Repeating Question | 171 | 0.92 | 91 | 0.65 | 94 | 0.66 | 62 | 0.42 | 56 | 0.36 |
| Fuzzy Question | 95 | 0.51 | 29 | 0.21 | 54 | 0.38 | 31 | 0.21 | 38 | 0.24 |
| Facilitative Communication | 1461 | 7.83 | 808 | 5.76 | 1296 | 9.05 | 1068 | 7.28 | 1059 | 6.79 |
| Off-topic Statement | 599 | 3.21 | 98 | 0.70 | 195 | 1.36 | 63 | 0.43 | 129 | 0.83 |
