# Supplementary material for: Developing and Validating a Coding Scheme for Clinical Reasoning in History Taking Using Generative AI–Based Virtual Patients: Systematic Text Condensation Approach
Source: JMIR Med Educ. 2026 Apr 13;12:e84347. doi: 10.2196/84347 (PMC13075466; doi:10.2196/84347)
Supplement: Multimedia Appendix 3 [file mededu-v12-e84347-s003.docx]

**Appendix 3: Sample of Student-Virtual Patient Dialog with Codes**

| **Student's Questions** | **Code** | **Patient's Responses** |
| --- | --- | --- |
| Hello, I'm your attending doctor. You can call me Dr. Zhang. | *Facilitative Communication* | Hello, Dr. Zhang. |
| Okay, where exactly do you feel uncomfortable? | *Routine Question* | I feel pain here in my chest. |
| Which specific area? | *Specifying Symptoms* | In the center of my chest. |
| Besides chest pain, do you feel pain elsewhere? | *Specifying Symptoms* | There's also pain in the left side of my chest. |
| How long has this symptom lasted? | *Specifying Symptoms* | It started last night and is still ongoing. |
| Could you think of any potential causes for your chest pain? | *Specifying Symptoms* | I just got back from a business trip. |
| On a scale of 0–10, how severe is your pain now? | *Specifying Symptoms* | Around 6 or 7. |
| What can relieve the pain? | *Specifying Symptoms* | Taking deep breaths or lying down helps. |
| Does it worsen with activity or deep breaths? | *Pathophysiologic Question* | Yes, it feels heavier when I move or breathe deeply. |
| Any symptoms like fever, cough, or shortness of breath? | *Logical Organization* | I've had a mild fever and occasional cough. |
| Any signs of leg swelling? | *Routine Question* | No swelling. |
| Did you visit any clinic before this? | *Routine Question* | This is my first visit. |
| Did you take any medicine or treatment before coming? | *Routine Question* | I took some painkillers, but it didn't have much effect. |
| Thank you for your understanding. | *Facilitative Communication* | I appreciate that, thank you. |
| You mentioned fever earlier, can you tell me more? | *Logical Organization, Specifying Symptoms* | It started three days ago, mostly at night. |
| Did you check your temperature? Any fluctuation? | *Logical Organization, Specifying Symptoms* | It was 38.7°C yesterday, still about the same now. |
| Have you been doing any physical activity lately? | *Routine Question* | I haven't exercised recently. |
| Two weeks ago, you developed a sore throat, runny nose, cough, and fever (temperature 37.8°C) due to catching a cold. About 8 hours ago, you gradually began experiencing pain in the middle of the left side of your chest, radiating to your left shoulder. It is a sharp pain, without a burning sensation, with an intensity around 7 out of 10. The pain is constant and does not come and go. It worsens with deep breathing and when lying flat, and there are no other identifiable factors that relieve or aggravate it... | *Summarizing & Integrating* | Doctor, your summary is mostly correct, but I still feel some chest pain. What exactly is wrong with me? Could it be a heart attack? I'm a bit scared. |
| Right now, we suspect it might be viral myocarditis, but we can't completely rule out a heart attack... | *Pathophysiologic Question* | Doctor, I'll fully cooperate. |
| What do you think caused your symptoms? | *Routine Question* | I'm not really sure. |
| Do you smoke? Any recent alcohol consumption? | *Routine Question* | I smoke, but haven't been drinking lately. |
| How many cigarettes a day? Ever thought of quitting? | *Specifying Symptoms, Routine Question* | About a pack a day. I want to quit. |
| Alright, drinking alcohol isn't beneficial to your health. As a doctor, I suggest you consider quitting alcohol to maintain your well-being. | *Facilitative Communication* | Thank you, I'll try. |
